# Understanding Dissolution Rates via Continuous Flow Systems with Physiologically Relevant Metal Ion Saturation in Lysosome

**DOI:** 10.3390/nano10020311

**Published:** 2020-02-12

**Authors:** Johannes G. Keller, Willie Peijnenburg, Kai Werle, Robert Landsiedel, Wendel Wohlleben

**Affiliations:** 1 BASF SE, Dept. Experimental Toxicology and Ecology and Dept. Advanced Materials Research, 67056 Ludwigshafen, Germany; johannes-georg.keller@basf.com (J.G.K.); kai.werle@basf.com (K.W.);; 2 Institute of Pharmacy, Faculty of Biology, Chemistry & Pharmacy, Freie Universität Berlin, 14195 Berlin, Germany; 3 National Institute of Public Health and the Environment RIVM, 3721 Bilthoven, The Netherlands; 4 Institute of Environmental Sciences (CML), Leiden University, 2333 Leiden, The Netherlands

**Keywords:** dissolution, dissolution rate, nanomaterial grouping, risk assessment, 3R method, regulatory hazard assessment

## Abstract

Dissolution rates of nanomaterials can be decisive for acute in vivo toxicity (via the released ions) and for biopersistence (of the remaining particles). Continuous flow systems (CFSs) can screen for both aspects, but operational parameters need to be adjusted to the specific physiological compartment, including local metal ion saturation. CFSs have two adjustable parameters: the volume flow-rate and the initial particle loading. Here we explore the pulmonary lysosomal dissolution of nanomaterials containing the metals Al, Ba, Zn, Cu over a wide range of volume flow-rates in a single experiment. We identify the ratio of particle surface area (SA) per volume flow-rate (SA/V) as critical parameter that superimposes all dissolution rates of the same material. Three complementary benchmark materials—ZnO (quick dissolution), TiO_2_ (very slow dissolution), and BaSO_4_ (partial dissolution)—consistently identify the SA/V range of 0.01 to 0.03 h/cm as predictive for lysosomal pulmonary biodissolution. We then apply the identified method to compare against non-nanoforms of the same substances and test aluminosilicates. For BaSO_4_ and TiO_2_, we find high similarity of the dissolution rates of their respective nanoform and non-nanoform, governed by the local ion solubility limit at relevant SA/V ranges. For aluminosilicates, we find high similarity of the dissolution rates of two Kaolin nanoforms but significant dissimilarity against Bentonite despite the similar composition.

## 1. Introduction

With a steadily increasing demand for engineered nanomaterials (ENMs) for industrial applications, the ENM production volumes reported by industry are a significant share of the total particle production [1]. The properties of the nanomaterials are specifically designed for their different intended uses and are therefore varied in surface treatment, composition, size, and morphology. Often one substance exists in many different nanoforms. The vast amount of nanoforms makes it impossible to investigate the risk potential of each individual nanoform [1,2]; therefore, grouping and read across approaches are urgently needed to reduce the uncertainty related to the risk of nanomaterials [3,4,5]. The nanoform is described through chemical composition, surface properties, size, and shape [6]. However, these properties are insufficient to forecast potential environmental and human health-related hazards and do not take into account the complex mechanism of pulmonary retention, clearance, and translocation that particles are exposed to upon inhalation [7,8,9,10]. Thus, the European Chemicals Agency (ECHA) as well as the Environmental Protection Agency (EPA) are prioritizing the dissolution rate as a key criterion for the assessment of the risk potential of nanomaterials [11]. This criterion can then be used to compare different nanoforms of a substance. Nevertheless, it is not sufficient to group the nanomaterials into soluble and insoluble [12] as the dissolution kinetic spans over many orders of magnitude from fast dissolving ENMs such as our benchmark materials ZnO and CuO to poorly soluble materials such as TiO_2_ and Fe_2_O_3_ [13]. The dissolution rate is measured in ion mass per solid particle surface per time and is designated as *k* [ng/cm²/h] [14]. In contrast, the solubility is measured in equilibrium saturated suspensions to yield the conventional solubility limit [4]. Low solubility should not be interpreted as a sufficient indication of biopersistence, because the static setup does not represent the conditions that particles are exposed to when being taken up into a physiological system. In comparison to the static system, the physiological system is an open system with both passive and active transport away from the remaining particles. The continuous flow-through system for nanomaterials is currently used for quantifying environmental [15] as well as oral dissolution [14,16] and was adapted from a system used for man-made vitreous fibers (MMVF) [17,18,19]. One might anticipate that the testing has to be done with a range of initial ENM mass concentrations at the same flow-rate in order to verify that the results are truly not limited by local concentrations reaching the solubility limit. Otherwise, a false negative result of apparently low dissolution rate might occur. The results on MMVF have highlighted that the solubility limit can be avoided by increasing the flow-rate at the same initial mass [20]. However, false positive results of overestimating the physiological ion transport and, hence, overestimating the biodissolution are also imaginable. Continuous flow systems (CFSs) have two adjustable parameters that can be used to prevent false predictions: the volume flow-rate and the initial particle loading. The decisive parameter in MMVF testing was the ratio SA/V of surface area per flow-rate, but this has not been explored yet for nanomaterials.

Here we propose and demonstrate a more efficient alternative to verify predictive conditions by ramping the fluid flow of the continuous flow system at the same initial ENM mass. The ramped flow-rates are applied to a set of nanoforms and non-nanoforms containing the critical metals Al, Ba, Zn, Cu with existing in vivo inhalation clearance results [12,21,22] to validate the dissolution results with three different benchmark materials for quickly soluble, soluble, and insoluble with ZnO, BaSO_4_, and TiO_2_, respectively.

## 2. Materials and Methods

The assessment of the dissolution rate of ENMs through flow-rate ramping is a complimenting technique to the current dissolution setup as described in Koltermann-Jülly et al. 2018 and Keller et al. 2020 [14,23]. The continuous flow-system was developed to assess the dissolution far from equilibrium. However, the solubility limit of the tested ENMs requires the tester to run multiple tests with varying initial masses in order to verify that the dissolution is not limited by local concentrations reaching the solubility limit. Therefore, the technique of flow-rate ramping is presented as an advanced technique that can be transferred to all materials and cannot fully be described by measuring at a single initial mass. The fundamentals of this testing methods are described in the next section.

Ten different materials were selected with high industrial relevance. Their basic nanoform descriptors and other properties have been published recently [24] and are reproduced in Table S1. These materials consist of two different kinds of BaSO_4_, one nanosized material provided by the Organization for Economic Co-operation and Development (OECD) sponsorship program and the non-nanosized BaSO_4_ IRMM381 as a non-nanosized form provided by the Institute for Reference Materials and Measurement (IRMM), with permission of Solvay [25,26]. CuO was produced by PlasmaChem GmbH, Germany [22]. Furthermore, two kinds of Kaolin IRMM-385 (JRC-IRMM, Geel) and Kaolin (BASF SE, Ludwigshafen) were used, which both differ in size and form and were provided by the OECD sponsorship program. Additionally, Bentonite NM600 was provided by the OECD sponsorship program. ZnO NM110 and NM111 were kindly provided by the Joint Research Center (JRC) repository of the OECD sponsorship program and differed in their surface coating. ZnO NM111 is used as a benchmark material for a quickly dissolving nanomaterial as per the available inhalation in vivo results and has a hydrophobic triethoxycaprylsilane coating [27,28].

### 2.1. Continuous Flow System with Programmable Sampling, ICP-OES or ICP-MS Analysis

**Figure 1:**
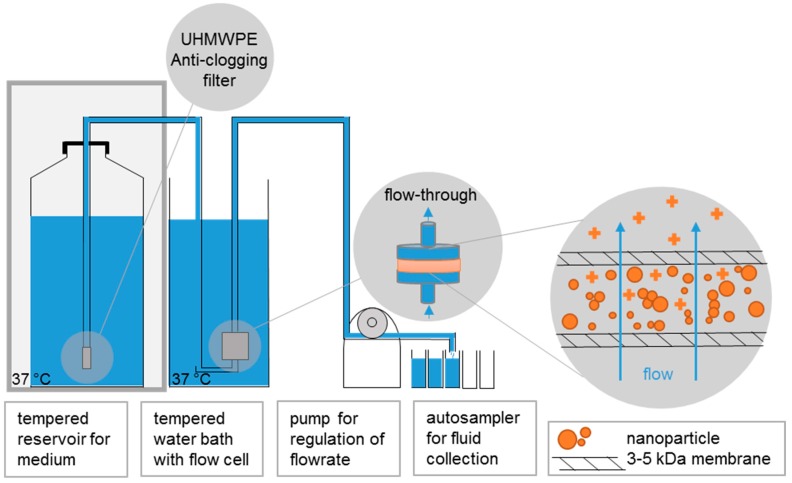
Dissolution setup. The reservoir for the physiological fluid was controlled at 37 °C, as well as the flow-through cells. The peristaltic pump regulated the flow-rate of up to eight cells in parallel, with a programmable autosampler for fluid collection. The flow-through cell was equipped with 5 kDa membranes to hold back particles and only allow the flow of ions. The meniscus of the reservoir was elevated approx. 0.5 m above the cells such that the hydrostatic pressure balanced the pressure drop by the 5 kDa membrane.

The setup (Figure 1) implements a “continuous flow system” described in ISO 19057:2017 [29]. It is essentially a replication of the established flow-through testing of the dissolution kinetics of mineral fibers [19,30], was described for this purpose [31], and was used here with minor adaptations to match the specifics of ENMs: The ENM mass of 1 mg was weighed onto a membrane (cellulose triacetate, Sartorius Stedim Biotech GmbH, Goettingen, Germany): 47 mm diameter, 5 kDa, and a pore size of ~1 nm, topped by another membrane, and enclosed in flow-through cells. Standard conditions are 1 mg initial solid mass in the flow-through cell, and 2 mL/h flow. The phagolysosomal simulant fluid (PSF) pH 4.5 (composition in Table S2), which is an acidic buffer simulating the phagolysosomal compartment of macrophages [31,32], and which was previously validated for the purpose of inhaled beryllium dissolution by National Institute of Standards and Technology (NIST) laboratories [32], was employed at 37 ± 0.5 °C. In contrast to the larger volume flow cells used by Bove et al. for oral dissolution testing [16], in our setup the ENMs were in direct contact with the ultrafiltration membrane. In contrast to the flow cells used by Kent et al., our cells held industrially produced ENMs, not lab-grown arrays of similar chemical composition [15]. The core idea of separating ions from remaining solids was shared by all three setups. The programmable sampler drew 4–10 mL of the eluate once to two times per day for seven days. Depending on the flow-rate (Table 1) the samples were drawn for 3–44 h per sample in order to fill the vials. The rest of the eluate was collected in a container. The ion concentrations of the eluates from different time points were determined by inductively coupled plasma–optical emission spectrometry (ICP-OES Agilent 5100 and Varian 725 ES) with a limit of detection of 0.1 mg/L, or alternatively by inductively coupled plasma–mass spectrometry (ICP-MS Perkin Elmer Nexion 2000b, Perkin Elmer Inc., Waltham, USA) with a limit of detection of 0.1 ppb. Prior to taking the measurement, the instrument was optimized in accordance with the manufacturer′s specification. Duplicate measurements were taken and averaged. We measured with 10 s integration time, and the dilution factors were between 1 and 10. External calibration used concentrations of 0/1/5 mg/L with matrix-matched standards. The nebulizer (Meinhard 1 mL) had a flow of 0.7 L/min at a pump rate of 15 rpm. For ICP-MS, duplicate measurements were taken and averaged. The eluate was diluted with a factor in between 100 and 1000 and the external calibration used concentrations of 0.1/1/10 ppb. The nebulizer had a flow of 0.92 L/min at a pump rate of 35 rpm.

### 2.2. Continuous Flow System with Ramped Flow-Rates to Ensure Out-Of-Equilibrium Conditions

To further understand the dissolution behavior of nanomaterials, the dissolution conditions were varied in order to ensure non-equilibrium conditions. This was done by varying the flow-rate drastically between 0.1 and 3 mL/h within a single measurement. The precise flow-rates can be seen in Table 1. The flow-rate was gradually changed after either each or every second filled vial. Therefore, the sampling time of a single time point was varied between 3 and 24 h. Besides the varying flow-rate, adjusted through the flow-regulating pump, the measurements were done in accordance to the continuous flow-through system with constant flow-rate. After each run, the ion content was determined through ICP-MS.

There are three ways to express the dissolution rate of the nanoparticles. These three ways have been described in our previous contribution [23], each working best to describe the dissolution mechanism of a certain set of materials and are reproduced here for completeness of methodology. For this purpose, we multiplied the amount of eluate with the ion concentration to determine the total ion mass dissolved, as illustrated for BaSO_4_:

- Cumulative rate: The amount of dissolved BaSO_4_ at each time point *M*_ion_(T) is expressed as a fraction of the initial mass loading (*M*_0_ = 100%) and cumulated from all samplings with concentration *c*_i_, flow *V*_i_, and sampling interval Δ*t*_i_, and includes the stoichiometry of BaSO_4_:
  (1) $$\frac{M_{ion}\left(T\right)}{M_{0}}=\frac{m\left(BaSO_{4}\right)}{m\left(Ba\right)*M_{0}}\sum_{i=0}^{T}c_{i}\left(Ba\right)V_{i}\Delta t_{i},$$
  (2) $$k=\frac{M_{ion}\left(t\right)}{M_{0}}\frac{1}{tBET}.$$
  The rate *k* incorporates the Brunauer–Emmett–Teller (BET) value [33]. The BET method uses adsorption of gases at constant temperature to determine the surface area of particles, in order to report results with a focus on composition or coating dependence, instead of size dependence. The conventional units of *k* are ng/cm²/h [30,34]. We typically determine *k* by the number of cumulated ions during a specific time interval at the end of the test.
- Curve fitting: To verify first-order dissolution kinetics [34], the cumulative dissolved BaSO_4_ mass is expressed as an inverse relationship, i.e., decreasing solid retained BaSO_4_ mass (*M*_ion_(*T*) – *M*_0_)/*M*_0_, and plotted against time on a semi-log scale. The dissolution rate—expressed as a fraction per hour —is calculated from the slope of this line and then converted to percent per day using the total system’s available starting mass. Dissolution rate and half-time (*t*’_1/2_, 50% dissolved) are inversely related and can be expressed in two alternative metrics (below) as given for first-order modeling in ISO 19057:2017 [29,34]. The BaSO_4_ dissolution half-time allows direct extrapolation and comparison to the in vivo dissolution t_1/2_ of inhaled BaSO_4_, which is derived from the total in vivo t_1/2_:
  (3) $$b_{diss}=\;\frac{\ln2}{t_{1/2}^{\prime }},$$
  (4) $$t\prime_{1/2}=\;\frac{\ln2}{b_{diss}}.$$
- Instantaneous rates: For each sampling interval Δ*t*, the instantaneous dissolution rate *k* was constructed as:
  (5) $$k\left(t\right)=\frac{\frac{M_{ion}\left(t\right)}{SA\left(t\right)}}{\Delta t},$$
  (6) $$SA\left(t\right)=BET\left(t_{0}\right)\left(M_{ion}\left(t\right)\right).$$
  We approximated the instantaneous surface area and, thus, ignored changes of the size distribution and shape (see Discussion). Elsewhere [14] we explored modeling of SA(*t*) via the assumption of shrinking spheres [29,34], which does not apply for particles with a tendency to undergo morphological transformation processes, such as BaSO_4_.

### 2.3. Terminology of Modeling Nanomaterial Dissolution

In this section, we recapitulate the relationship between the ENM, assumed to be a spherical particle, and the ion concentration. For this, we introduce the following notation:

- R(T): radius of a particle in cm with $R_{0}=R\left(0\right)$
- $v\left(t\right)=\frac{4}{3}\pi R^{3}$: volume of a particle
- $A\left(t\right)=4\pi R^{2}$: area of a particle
- k: rate constant at which particle dissolves in ng/cm²/h
- ρ: density of particle g/cm³
- t: time in h
- I(t): ions present in the solution in g
- m: mass of fluid in the flow cell, in g
- $\dot{m}$: flow of fluid per time, in g/h.

Then when extending the model to a suspension of particles we use

- $C_{NP}\left(t\right)$: concentration of particles at time *t* in g/L
- $C_{ion}\left(t\right)$: concentration of ions at time *t* in g/L
- $N_{0}$: number of NPs present in the solution per liter at t=0 in 1/L.

In this notation, the concentration of particles (NP) and ions are given by

(7) $$C_{NP}\left(t\right)=N_{o}v\left(t\right),$$

(8) $$C_{ion}\left(t\right)=N_{o}I\left(t\right).$$

Due to dissolution, the well-established “shrinking sphere” model [35] links the generation of ions to a reduction of the particle volume:

(9) $$\frac{dC_{NP}}{dt}=N_{0}\rho \frac{dv}{dt}=-kAN_{0}=-k4\pi R^{2}N_{0},$$

(10) $$\frac{dC_{ion}}{dt}=N_{0}\frac{dI}{dt}=kAN_{0}=k4\pi R^{2}N_{0}.$$

## 3. Results

**Figure 2:**
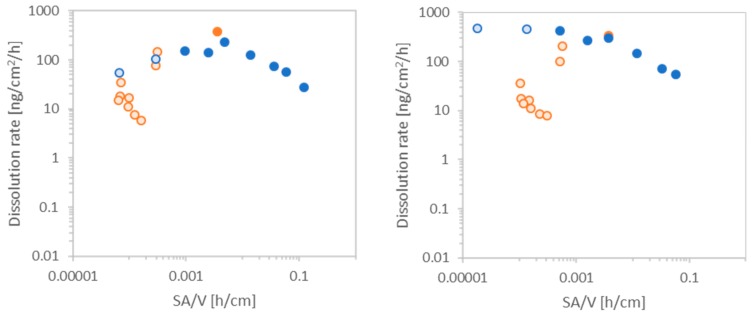
Time-resolved dissolution kinetics of CuO (left) and ZnO NM 111 (right). The blue curve indicates the ascending flow-rate ramping, whereas the orange curve indicates a descending flow-rate ramping. Unfilled circles indicate points with remaining mass calculated to be <10%; the corresponding instantaneous rates are equally marked not sufficiently reliable in Figure 3.

**Figure 3:**
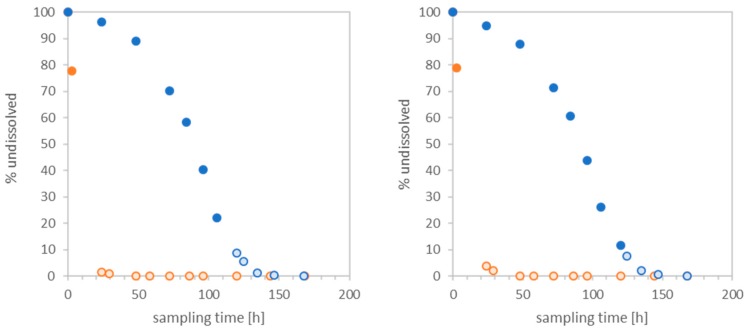
Dissolution rate depending on surface area and flow-rate. CuO (left) and ZnO NM111 (right). Blue curve indicates ascending and orange curve descending flow-rate ramping. Unfilled circles indicate points with low reliability due to remaining mass <10%. Note the superimposed orange and blue dots.

Nine nanomaterials and one particle of bigger size were tested within the flow-through dissolution system with ramped flow-rates. The dissolution of the nanomaterials in phagolysosomal simulant fluid at pH 4.5 with increasing flow-rate was compared against a run with decreasing flow-rate as shown in Figure 2. The blue data points display the ascending flow-rate ramp from 0.1 to 3 mL, whereas orange dots indicate the descending flow-rate from 3 to 0.1 mL/h. Similar time-resolved dissolution curves were obtained for the nanomaterials ZnO NM111 and CuO. Both materials dissolve completely after a time period of 7 days either with the ramp up or the ramp down, considering that the acidic conditions of pH 4.5 are the driving force of this complete dissolution. Both materials could easily be measured with the He kinetic energy discrimination (KED) mode [35,36] of the ICP-MS since their limit of detection (LoD) is rather low, even traces up to 0.01 ppb could be measured. However, despite their similarity, the difference between the ascending and descending curve is significant. Interestingly, the ascending flow-rate forms an S-shaped curve while the descending flow-rate describes a rapid decrease of nanomaterial and after less than 50 h 100% of the nanomaterial is already dissolved. Thus, the descending flow-rate ramp cannot be used to better describe the dissolution for quickly dissolving nanomaterials. At the beginning phase of the ascending flow-through measurement, a high initial mass, which goes along with high surface area is exposed to a low flow-rate, resulting in a slower dissolution. The data points for the ascending flow-rate ramping are spread over a broader distribution of surface area to flow-rate (Figure 3). These graphs also prove that the measurement was done under out-of-equilibrium conditions and the saturation limit of the ions was not reached. Upon reaching saturation, data points are expected to be in a straight line.

**Figure 4:**
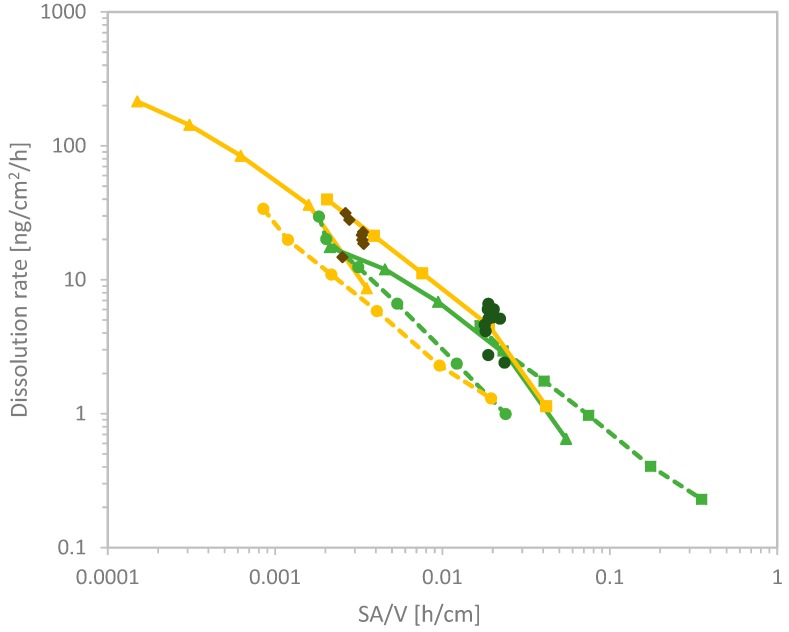
Dissolution rate of BaSO_4_ NM220 (green) and BaSO_4_ IRMM381 (yellow). The SA/V is plotted against the dissolution rate *k* [ng/cm²/h]. The dashed line indicates the ramp down with decreasing flow-rate 3–0.1 mL/h and the solid line represents the ramp up with increasing flow-rate from 0.1 to 3 mL/h. The two different dashed green curves vary in initial mass. The dashed green curve with the square has an initial mass of 1.01 mg, whereas the curve with the round symbol has an initial mass of 0.14 mg. The two yellow solid curves vary from 1.42 mg initial mass for the square to 0.12 mg for the triangle. Both darker spots represent the dissolution rate for the dynamic dissolution with constant flow-rate for nano BaSO_4_ NM220 yellow and BaSO_4_ IRMM381 dark green.

The results on the dissolution with ramped flow-rate on two different BaSO_4_ particles, BaSO_4_ NM220 and BaSO_4_ non-nano are displayed in Figure 4. Two initial masses for the ramp down (dashed line) of BaSO_4_ NM220 (green) and one for BaSO_4_ IRMM381 (yellow) were compared to the ramp up (solid line) for one initial mass of BaSO_4_ NM220 and two initial masses of BaSO_4_ IRMM381. The graph shows the plot of SA/V versus the dissolution rate *k* with dissolution rates that differ by a few orders of magnitude. This difference can be due to the difference in BET, as for instance the nano BaSO_4_ possess a 16-fold higher surface area than the non-nano form. Furthermore, this observation is confirmed by the time-resolved dissolution curve as seen on the left-hand side of Figure 4. However, when normalizing the surface area with different initial masses for the ramp up (BaSO_4_ NM220 0.11 mg PSF vs. BaSO_4_ IRMM381 1.42 mg PSF) as well as for the ramp down (BaSO_4_ NM220 0.14 mg PSF vs. BaSO_4_ IRMM381 0.86 mg), similar values for the dissolution rate can be observed for the two different nanoforms. Therefore, the results for the increasing flow-rate as well as the results for the static flow-rate do achieve a good predictability for dissolution rates between 0.01 and 0.03 h/cm; whereas the decreasing flow-rate as seen in Figure 4 cannot be applied.

The consistency between the instantaneous dissolution rates determined with fixed flow vs. ramped (increasing) flow has been assessed for BaSO_4_ NM220, CuO, ZnO NM110, and ZnO NM111, and the results in the SI (Figure S4) demonstrate a) that the rule of 10% remaining mass successfully removes unreliable data and b) that the ramped flow enlarges the range of SA/V that is accessible in a single measurement. Data below 10% remaining mass are considered unreliable since weighing in 1 mg of ENM into the flow-through cell always induces uncertainties. Furthermore, by calculating the dissolved fraction for quickly dissolving materials, often no ions can be detected anymore even though dissolution has not reached 100% yet.

The apparent inverse linear relationship between the dissolution rate and SA/V was found for BaSO_4_ NM220 before [23], but the new finding that this behavior extends to the non-nanoform and across a wide range of volume flow-rates (Figure 4) deserves a mechanistic understanding. Here we develop a model of partial and very slow dissolution in the continuous flow system, where a fraction of the fluid mass $\dot{m}/m$ is exchanged during each time interval. To represent the influence of saturation, $f\left(C_{ion}\right)$ is a function that decays to zero at the ion solubility limit, and *f* (0) = 1:

(11) $$\frac{dC_{ion}}{dt}=N_{0}\frac{dI}{dt}=4k\pi R{\left(t\right)}^{2}N_{0}f\left(C_{ion}\right).$$

Compared to Equation (9), the saturation term in Equation (11) limits the ion concentration, where the curve shape of $f\left(C_{ion}\right)$ of course depends on substance and medium. Importantly, the flow introduces an additional loss term to the ions (not to the particles, due to the ultrafiltration membrane!), leading from Equation (11) to Equation (12):

(12) $$\frac{dC_{ion}}{dt}=N_{0}\frac{dI}{dt}-N_{0}I\frac{\dot{m}}{m}=4k\pi R{\left(t\right)}^{2}N_{0}f\left(C_{ion}\right)-N_{0}I\frac{\dot{m}}{m}.$$

This assumes that the ion concentration is homogeneous in the volume of the flow cell. We can now introduce the experimental observation that, for partial and very slow dissolution, the concentration of ions $C_{ion}$ does not change over time: $\frac{dC_{ion}}{dt}=0$, and obtain:

(13) $$I\frac{\dot{m}}{m}N_{0}=4k\pi R{\left(t\right)}^{2}N_{0}f\left(C_{ion}\right).$$

Using the notation from MMVF literature $SA\left(t\right)∶=4\pi R{\left(t\right)}^{2}$ and the volume flow-rate $V_{fluid}∶=\dot{m}\;/\rho_{fluid}$ this expression transforms to

(14) $$I\frac{\rho_{fluid}V}{m}=k\;SA\left(t\right)\;f\left(C_{ion}\right).$$

Solving for *k*, and simplifying $C_{ion}=I\frac{\rho_{fluid}}{m}$, we obtain

(15) $$k=\frac{C_{ion}}{f\left(C_{ion}\right)}\frac{V}{SA\left(t\right)}.$$

Hence the measured *k* is reduced by saturation and is inversely proportional to the SA/V ratio with:

(16) $$k\propto \frac{1}{SA/V}.$$

**Figure 5:**
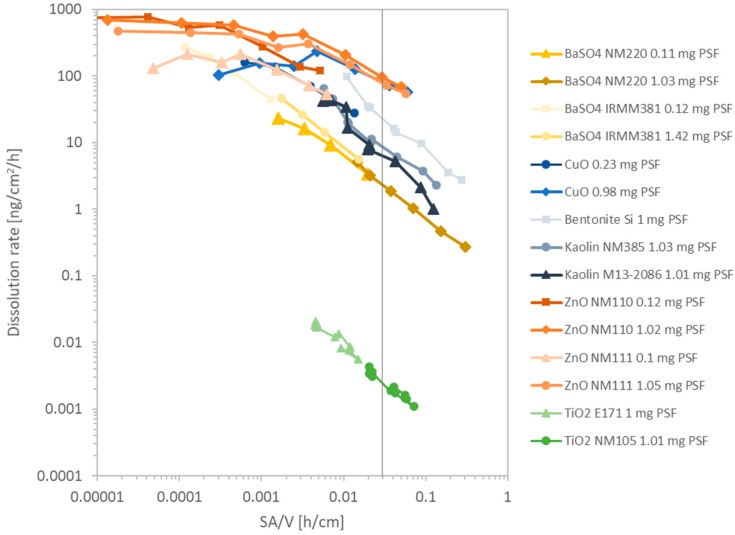
Cumulated SA/V plot vs. dissolution rate *k* [ng/cm²/h] of 10 different tested nanomaterials in 15 different runs with varying initial masses. The dots of each run are connected through a line with each other for visualization purpose only. The family of TiO_2_ (nano)forms is displayed in green, BaSO_4_ (nano)forms in yellow, CuO in blue, ZnO in shades of orange, and aluminosilicates Bentonite and Kaolin in grey. The vertical line displays the optimum SA/V.

As a next step, we explored whether this universal relationship applies to partial and very slow dissolution of substances other than BaSO_4_. In total, 15 different flow-through dissolution measurements with an increasing flow-rate were conducted and combined within Figure 5. The dissolution rate of the quickly dissolving benchmark materials, CuO as well as three different kinds of ZnO were compared with moderately soluble Kaolin, Bentonite, BaSO_4_, and insoluble nanoforms of TiO_2_ NM105 and food grade non-nano TiO_2_ E171. This graph especially shows that there is a significant difference of the dissolution rate between the quickly dissolving nanoforms of ZnO and CuO and the moderately soluble nanomaterials. All quickly dissolving materials exhibited an almost horizontal line in the graph which represents a continuous dissolution rate, which is not dependent on the surface area of the nanomaterial. Nevertheless, ZnO NM110 shows a significantly slower dissolution for certain flow-rates and therefore high SA/V values must be excluded. The graph shows that even quickly dissolving materials such as ZnO can show dissolution rates below 100 ng/cm^2^/h if the dissolution is tested at SA/V values below 0.1 h/cm. However, the plot additionally shows diagonal curves for BaSO_4_ NM220, BaSO_4_ IRMM381, Kaolin JRC-IRMM385, Kaolin, and Bentonite.

Both materials, ZnO NM110 and the surface-treated ZnO NM111 exhibited a similar dissolution process. Therefore, a significant impact of the hydrophobic triethoxycaprylsilane coating could not be observed. Furthermore, Bentonite as well as both Kaolin materials showed a similar dissolution behavior with a diagonal line in the SA/V vs. *k* plot. The comparison of different dissolution rates of aluminosilicates depending on the analyzed ion is shown in Figure S3. The graphs S3A–C confirm the hypothesis that Si dissolves more readily than Al, with a factor of 100 between the respective dissolution rates. Therefore, the dissolution of Al is the rate-limiting step in the dissolution kinetics of aluminosilicates.

The two insoluble materials, TiO_2_ E171 and TiO_2_ NM105, furthermore show a diagonal slope in Figure 5 just like the other partially soluble materials. This behavior is understood and explained in the previous mentioned equations. However, when both TiO_2_ particles were tested at low SA/V values below 0.001 h/cm false positive predictivities were observed which are not in agreement with observations made in in vivo studies [37], nor other previous dissolution experiments. Therefore, for the test, the flow-rate must ne adjusted tp keep SA/V above 0.001 h/cm. The quickly soluble ENMs, CuO and ZnO, exhibited horizontal lines within the SA/V against *k* plot at low SA/V ratios below 0.001 h/cm.

Both flow-rate operation modes (fixed and ramped) are compared against each (Figure S4). Here similar initial masses of BaSO_4_ NM220, CuO, ZnO NM110, and ZnO NM111 were measured for both models. The flow-rate ramp with increasing flow increases the spread of the data points more than done by the fixed flow. This results in better spread data points of the dissolution. This characteristic can be best observed for BaSO_4_ NM220 in Figure S4A, where a cloud of data points is achieved through fixed flow-rate dissolution.

## 4. Discussion

### 4.1. Materials

Only the two materials, CuO and ZnO, which are expected to be quickly dissolving and not biopersistent, reached close-to-horizontal lines in the *k* vs. SA/V plot (Figure 5), indicative of no further influence of the fluid flow velocity on dissolution, and hence the complete absence of local saturation. However, for CuO and ZnO also, local saturation at the solubility limit can occur, and for values of SA/V above 0.1 h/cm, the dissolution rate of both materials drops below 100 ng/cm²/h, and rates corresponding to halftimes longer than two days are predicted. Since in vivo clearance of Zn und Cu happens within a short period of few days, the prediction based on *k* values must not drop below 100 ng/cm²/h. This finding is therefore not in agreement with in vivo clearance of ZnO within days [22,38], and a dissolution test performed at this ratio is not predictive of the in vivo biopersistence. SA/V ratios above 0.1 h/cm therefore need to be excluded.

On the other end of the biopersistence range, even insoluble materials such as both TiO_2_ (nano)forms can be pushed under artificial conditions to show partial dissolution (above 0.1 ng/cm²/h, corresponding to halftimes below 500 days) when tested at SA/V ratios below 0.001 h/cm. TiO_2_ clearance after inhalation is dominated by physical transport within 40–70 days, not by dissolution [39,40,41,42,43,44]. Hence, measurements with a dissolution halftime faster than a few weeks, corresponding to *k* > 0.1 ng/cm²/h, are not in agreement with in vivo data [39,40,41,42,43,44]. Thus, a test at this ratio is not predictive of the in vivo biopersistence and SA/V ratios below 0.001 h/cm need to be excluded on the basis of TiO_2_ data.

In the intermediate range of partially dissolving materials, even though non-nano BaSO_4_ possess higher dissolution rates than the nanosized BaSO_4_ NM220 as shown in Figure 5, the nanoform still dissolves quicker under the same physiological conditions due to the increased BET surface, which is the link between dissolution rate and halftime. Figure 4 shows the similarity of the dissolution rate *k* between the nanoform BaSO_4_ NM220 and the non-nanoform BaSO_4_ IRMM381. Despite their different particle sizes, both materials exhibited similar dissolution rates when normalized to their surface area. No matter in which direction the flow-rate ramping was set to, either up or down, the dissolution rate remained similar between the non-nanoform and the nanoform. Both forms exhibited a diagonal trend. Thus, the ramped measurement confirms that the dissolution of BaSO_4_ of any size is limited by local ion concentration. The ion solubility limit is the rate-limiting factor, which is a property of the ion and is independent of the particle size. These findings are in strong agreement with the modeling developed here.

Aluminosilicates are a challenge for risk assessment, because the chemical sum formula places them in the substance class of silicates, but the physical structures and the bio-interaction are distinctly different from that of pure silicates: Studies on mineral fibers established that at acidic pH the mixed Si–Al-oxides have a significantly higher dissolution rate than pure Si-oxides [30,45]. Synthetic amorphous silica was recently re-evaluated by the Scientific Committee on Consumer Safety (SCCS), finding them “insoluble” [46] with insufficient toxicological evidence to assess the risk by human ingestion [47]. Furthermore, the platelet shapes of Kaolin (Al_2_Si_2_O_5_ (OH)_4_) or Bentonite (Al_2_H_2_Na_2_O_13_Si_4_) may induce unusual biological interactions via the opposite charges of lateral and edge surfaces. The widespread use of Bentonite as clarifying agent in wine making highlights its high protein binding capacity [48]. However, also when biodissolution leads to reduced biopersistence, the release of Al^3+^ ions raises concerns, as highlighted by the recent considerations of ECHA on read-across of aluminum salts [49]. Bentonite dissolves quicker than its two different Kaolin (nano)forms. Nevertheless, both Kaolin forms do show a similar behavior considering both Si and Al dissolution. In contrast, the Al of Bentonite is released at a threefold higher dissolution rate and is thus more bioavailable. Hence, the remaining structure of the aluminosilicates is enriched in Al and should further be assessed by in vitro as well as morphological approaches such as transmission electron microscopy (TEM).

### 4.2. Method

The modeling was based on the well-known “shrinking sphere” assumption [34], but added a loss term of ion transport through the ultrafiltration membrane of the flow cell, and added a saturation limit of ion concentration. The model provided an analytical basis (Equation 16) for the inverse linear relationship between *k* and SA/V that was observed for all materials (BaSO_4_ (nano and non-nano), TiO_2_ (nano and non-nano), Bentonite, Kaolin) as soon as saturation contributes:

- The model confirms that the prefactor is specific to the solubility limit of the ions in the medium, but independent of the size *R* of the particles.
- The model confirms that the slope of the inverse relationship to SA/V is universal for all substances, all sizes thereof, and all medium compositions. This aspect is proven here with experimental data on pulmonary lysosomal dissolution but is predicted as well for gastro-intestinal dissolution.

The screening of the different flow-rates within the dynamic flow-through dissolution setup with ramped flow-rates shows that flow-rates can be adapted in order to optimize the predictivity of the dissolution of nanomaterials. The advancements of the ramped flow-rate compared to the fixed flow-rate are clearly visible for BaSO_4_ NM220 (Figure S4A). Here the fixed flow-rate leads to a cloud of datapoints, whereas through the ramped flow the datapoints are more widespread, which leads to a better understanding of the dissolution mechanism. This observation was also made for both ZnO NM110 and NM111. Especially for these two quickly dissolving materials, an improvement of the datapoints could be achieved. Nevertheless, this increase is not as significant for the fast dissolving ENM CuO (Figure S4B).

The aim of this setup was to match the dissolution rate of nanomaterials with the clearance measured within in vivo systems. Thus, through increasing or decreasing the flow-rate by a factor of 100 the dissolution kinetics can be matched with the clearance in the rat lung. Though we observed false predictivities for some flow-rates (Figure 5), there were some flow-rates which led to a slower dissolution for ZnO nanoparticles but furthermore also to an increased dissolution of TiO_2_. These false predictivities can be excluded by sticking to a certain SA/V range between 0.01 and 0.03 h/cm. Within this SA/V range (Figure 5), the predicted biodissolution matches existing in vivo expectations: ZnO is cleared from lungs within days, whereas clearance of TiO_2_ is dominated by physical transport, not by dissolution [14,22,28]. For BaSO_4_ NM220, the relatively fast in vivo clearance with halftime of 9.6 days was attributed to partial dissolution with 11.1 days halftime [23,50]. With the BET of NM220, this halftime corresponds to *k* = 6.3 ng/cm²/h, which is obtained at SA/V = 0.02 h/cm (Figure 5). We thus recommend the SA/V range of 0.01–0.03 h/cm for optimal predictivity of biopersistence under pulmonary lysosomal conditions. This value is consistent with all benchmark materials.

However, based on the results seen in Figure 2, the ramp with decreasing flow-rate is not suitable for quickly dissolving materials and of limited reliability for the case of partially dissolving materials such as both BaSO_4_ (nano)forms.

Even though the flow-through system is an advanced system for the analysis of the dissolution of nanomaterials there are still some drawbacks which could not be fixed until now. One of the major reasons of measurement uncertainties of quickly dissolving ENMs is the void volume of the flow-through cell. The cell consists of three different stages which are separated from each other through two 5 kDa membranes, the dissolution happens in stage two, where the medium interacts with the ENM. After dissolution the ions penetrate the membrane into stage three and are then transported away and sampled into vials. However, the first 3 mL of the measurement that are sampled into the vials are part of the void volume of the system and did not interacted with the nanomaterials yet. Therefore, these measurements can be discarded. Thus, in order to minimize errors, only samples after at least a volume exchange by a factor of two are used for calculations.

Another reason for uncertainty arises from the interpolation of the remaining solids. Even though we created two different calculation systems for the dissolution process (“shrinking spheres” and “vanishing particles”) the dissolution of an ENM cannot fully be described by either one of these processes but might also be influenced by a combination of both scenarios. This calculation influences both the dissolution rate as well as the remaining mass. With decreasing remaining solid and increasing flow-rate the dissolution-rate, *k* increases significantly. This increase does not represent the median dissolution-rate and is often 2–4 times higher. Therefore, values with soluble ENMs with less than 10% remaining solid at any point in time were discarded and neither used for plots nor for further calculations.

## 5. Conclusions

Ramping the flow-rate is a promising approach to expand the range of regular out-of-equilibrium flow-through dissolution to nearly five orders of magnitude of the decisive parameter SA/V, which is the ratio of surface area and volume flow. This is especially relevant for slowly or partially dissolving ENM where the dissolution can be limited by saturation conditions. A model predicted a universal scaling of dissolution rates with SA/V and was confirmed by data on ENM dissolving to Al, Zn, Cu, Ba, Ti ions. The ramping allows the operator to investigate the dissolution properties within one single run and one initial mass which usually needs two to three different masses to fully describe the dissolution. The ramp with *increasing* flow-rate is useful as a screening tool for quickly dissolving ENMs because the wide range of flow-rates produces more relevant data points before complete dissolution compared to flow-through dissolution with fixed flow. The proposed benchmark materials for quick dissolution under acidic conditions—CuO, ZnO NM110—and the benchmark for very slow dissolution—TiO_2_ E171 and NM105—are confirmed as suitable benchmark materials, and are decisive to identify appropriate ranges of fluid flow velocities (V) and SA/V ratios: Extremes of SA/V below 0.001 h/cm or above 0.1 h/cm are excluded by the calibration of the predicted biopersistence against the in vivo observed pulmonary biopersistence. The additional case of the partially dissolving material, BaSO_4_, allowed us to recommend the range 0.01–0.03 h/cm for optimal predictivity of biopersistence under pulmonary lysosomal conditions.

For the cases studied here, TiO_2_ and BaSO_4_, the nanoform and the non-nanoform had the same dissolution rate normalized to the specific surface area. For Kaolin, different nanoforms of the same substance had the same dissolution rate. For all aluminosilicates, significantly faster leaching of Si than of Al was observed, leading to the prediction of crystalline structure transformation, and faster release of ions from Bentonite than from Kaolin. The diagonal, in the *k vs* (SA/V) plot, as reported earlier for BaSO_4_ NM220 [50], is not a unique phenomenon but is observed for all materials with partial dissolution limited by local ion solubility limits, even for (nano)forms of TiO_2_. The specific rates of the BaSO_4_ NM220 nanoform were also observed for the non-nanoform BaSO_4_ IRMM381. The dissolution of both materials is therefore governed by the same mechanism and by the same solubility limit.

## Figures and Tables

**Table 1:** Ramped flow-rates in dependence of time. Ramp up describes an increasing flow-rate from 0.1 to 3 mL/h, and ramp down describes a decreasing flow-rate from 3 to 0.1 mL/h.

	Flow-Rate [mL/h]	
Sampling Time [h]	Ramp Up ↑	Ramp Down ↓
24	0.1	3.0
48	0.1	3.0
72	0.2	2.0
84	0.5	2.0
96	0.5	1.0
106	1.0	1.0
120	1.0	0.5
125	2.0	0.5
135	2.0	0.2
147	3.0	0.1
168	3.0	0.1

## Supplementary Materials

The following materials are available online at https://www.mdpi.com/2079-4991/10/2/311/s1: Table S1: Descriptors and properties of tested engineered nanomaterials. Reproduced from Wohlleben et al. (Nanoscale, 2019). Table S2: Chemical composition of phagolysosomal simulant fluid (PSF) as reproduced from Keller et al. (Sci. Rep., 2020). Figure S3: Dissolution kinetic of three different Aluminosilicates. Grey indicates the dissolution of Si ions, whereas red indicates the dissolution kinetic of Al ions. Bentonite NM600, Kaolin JRC-IRMM385, and Kaolin 1 in Figure S3A–C respectively. Figure S4: Comparison of SA/V vs. dissolution rate *k* between the fixed flow-rate dissolution setup (orange) and the ramped flow-rate dissolution setup (blue) for **A** BaSO_4_ NM220, **B** CuO, **C** ZnO NM110, and **D** ZnO NM111. Unfilled circles indicate points with low reliability due to remaining mass <10%.
