# Supplementary material for: Understanding Dissolution Rates via Continuous Flow Systems with Physiologically Relevant Metal Ion Saturation in Lysosome
Source: Nanomaterials (Basel). 2020 Feb 12;10(2):311. doi: 10.3390/nano10020311 (PMC7075195; doi:10.3390/nano10020311)
Supplement: Supplementary file 1 [file nanomaterials-10-00311-s001.pdf]

Understanding dissolution rates via continuous flow systems with physiologically relevant metal ion saturation in lysosome – Supplementary Material

Johannes Keller[1,2], Willie Peijnenburg[3,4], Kai Werle[1], Robert Landsiedel[1], and Wendel Wohlleben [1,*]

[1] BASF SE, Dept. Experimental Toxicology and Ecology and Dept. Advanced Materials Research, 67056 Ludwigshafen, Germany; johannes-georg.keller@basf.com, kai.werle@basf.com

[2] Institute of Pharmacy, Faculty of Biology, Chemistry & Pharmacy, Freie Universität Berlin, 14195, Berlin, Germany

[3] National Institute of Public Health and the Environment RIVM, P.O. Box 1, Bilthoven, The Netherlands; willie.peijnenburg@rivm.nl

[4] Institute of Environmental Sciences (CML), Leiden University, PO Box 9518, Leiden, The Netherlands

* Correspondence: wendel.wohlleben@basf.com; Tel.: +49-621-609-5339

Table S1: Descriptors and properties of tested engineered nanomaterials. Reproduced from Wohlleben et al. (**Nanoscale, 2019**).

	**descriptors**	**NM110_ZnO**	**NM111_ZnO**	**CuO**	**NM105_TiO2_nano**	**TiO2_non-nano**	**NM600_Bentonit**	**IRMM385_Kaolin**	**Kaolin**	**IRMM381_BaSO4 non-nano**	**IRMM387_NM220_BaSO4**
**Composition**	CAS no.	1314-13-2		1317-38-0	1317-80-2		1302-78-9	1332-58-7	1332-58-7	7727-43-7	7727-43-7
**Primary particle dimension**	median diameter, nm	42	80	24	21	204	1362	181	279	234	32
**Specific surface area (BET/VSSA)**	BET, m^2/g	12	14	34	51	15	52	16	24	2.5	41
	% C	30	67.9	7	9						17
	% O	38	24.3	47	63						52
	% metals	38	4.3	46	29						13
	% non metals	3	3.5								17
**Surface charge (zeta-potential)**	Zeta potential at pH7, mV	30	-25	-34	-17	36	-31		-53	-37	-37
**Hydrophobicity**	water contact angle	10	152	10	60	10	10	10	10	10	10
**Solubility in water**	OECD screening LoD or value, metal ion, ppm	3.6	3.3	97	ns	ns	0.1		0.8		6

Table S2: Chemical composition of phagolysosomal simulant fluid (PSF) as reproduced from Keller et al. (**Sci. Rep., 2020**)

**Substance**	**PSF pH4.5**
NaCl	6650
Na_2HPO_4	142
Na_2SO_4	71
$CaCl_2$ $2H_2O$	29
KH-Phthalate	4085
Glycine	450
alkylbenzyldimethylammonium chloride (ABDC)	50

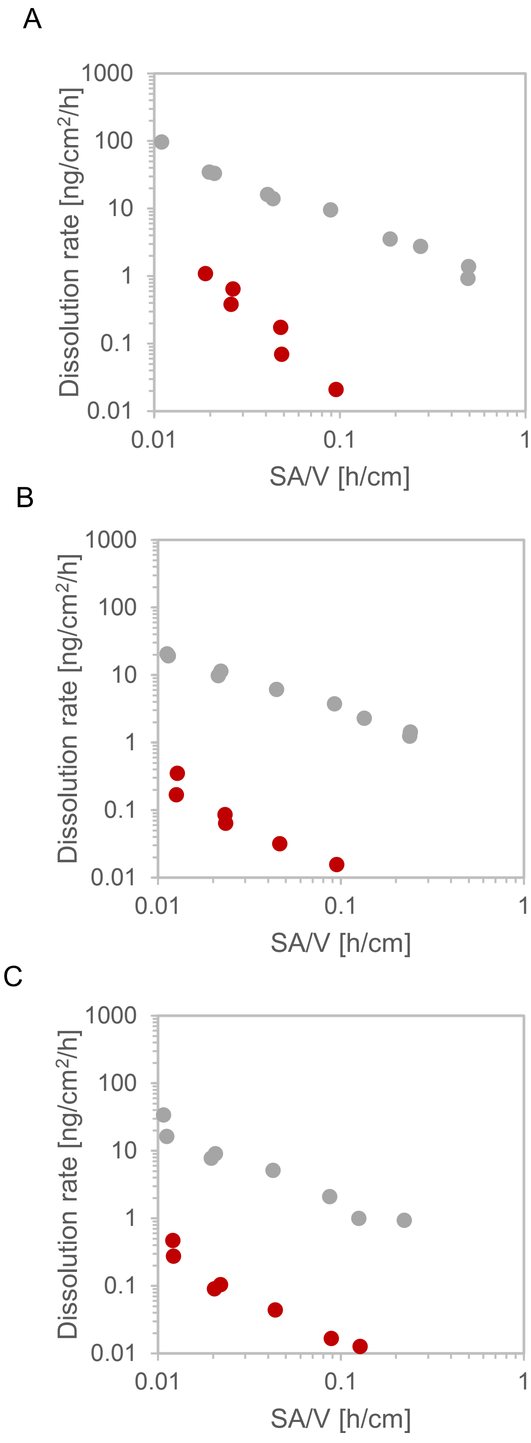

Figure S3: Dissolution kinetic of three different Aluminosilicates. Grey indicates the dissolution of Si ions, whereas red indicates the dissolution kinetic of Al ions. A) Bentonit NM600, B) Kaolin JRC-IRMM385, C) Kaolin (from nanoGRAVUR).

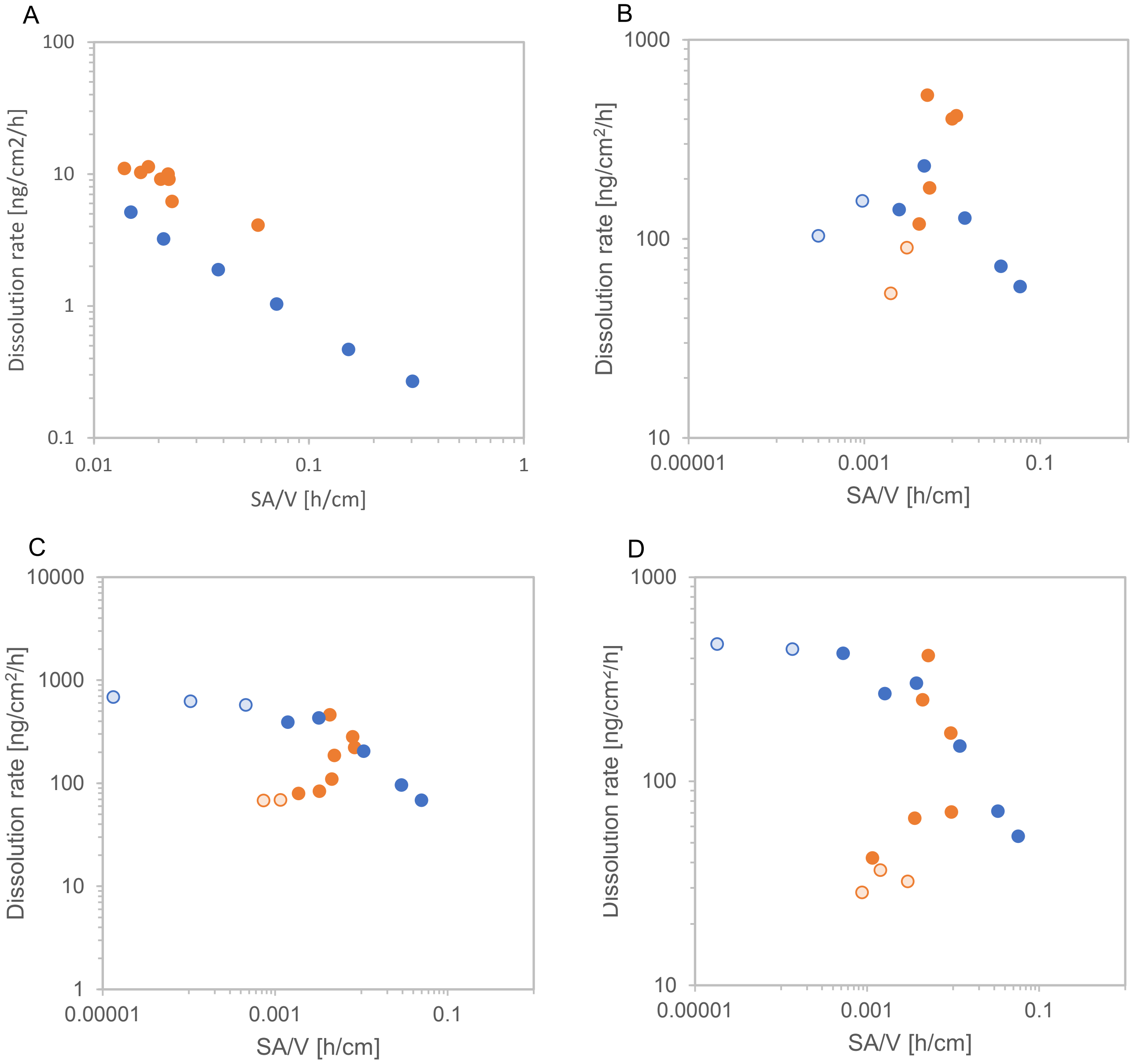

Figure S3: Comparison of SA/V vs. dissolution rate k between the fixed flow-rate dissolution setup (orange) and the ramped flow-rate dissolution setup (blue) for **A** $BaSO_4$ NM220, **B** CuO, **C** ZnO NM110 and **D** ZnO NM111. Unfilled circles indicate points with low reliability due to remaining mass <10%.
